# Perspectives on neurological patient registries: a literature review and focus group study

**DOI:** 10.1186/1471-2288-13-135

**Published:** 2013-11-09

**Authors:** Lawrence Korngut, Gail MacKean, Lisa Casselman, Megan Johnston, Lundy Day, Darren Lam, Diane Lorenzetti, Janet Warner, Nathalie Jetté, Tamara Pringsheim

**Affiliations:** 1 Department of Clinical Neurosciences and Hotchkiss Brain Institute, University of Calgary, Clinical Neurosciences, South Health Campus, 4448 Front Street SE, Calgary, Alberta T3M 1M4, Canada; 2 Department of Community Health Sciences, Faculty of Medicine, University of Calgary, TRW Building, 3rd Floor, 3280 Hospital Drive NW, Calgary, Alberta T2N 4Z6, Canada; 3 Research, Planning and Engagement, 5208 Veronica Rd NW, Calgary, Canada; 4 Department of Community Health Sciences, University of Calgary, 3rd Floor, TRW, 3280 Hospital Drive NW, Calgary, Alberta T2N 4Z6, Canada; 5 Department of Clinical Neurosciences and Hotchkiss Brain Institute, University of Calgary, 1403 29 Street NW, Calgary, Alberta T2N 2T9, Canada; 6 Department of Community Health Sciences and Institute for Public Health, University of Calgary, 1403 29 Street NW, Calgary, Alberta T2N 2T9, Canada; 7 Department of Clinical Neurosciences, Psychiatry, Pediatrics and Community Health Sciences, and Alberta Children’s Hospital Research Institute, University of Calgary, Alberta Children’s Hospital, 2888 Shaganappi Trail NW, Calgary, Alberta T3B 6A8, Canada

**Keywords:** Patient registries, Perspectives, Neurology, Focus group, Review

## Abstract

**Background:**

Patient registries represent a well-established methodology for prospective data collection with a wide array of applications for clinical research and health care administration. An examination and synthesis of registry stakeholder perspectives has not been previously reported in the literature.

**Methods:**

To inform the development of future neurological registries we examined stakeholder perspectives about such registries through a literature review followed by 3 focus groups comprised of a total of 15 neurological patients and 12 caregivers.

**Results:**

(1) Literature review: We identified 6,435 abstracts after duplicates were removed. Of these, 410 articles underwent full text review with 24 deemed relevant to perspectives about neurological and non-neurological registries and were included in the final synthesis. From a patient perspective the literature supports altruism, responsible use of data and advancement of research, among others, as motivating factors for participating in a patient registry. Barriers to participation included concerns about privacy and participant burden (i.e. extra clinic visits and associated costs). (2) Focus groups: The focus groups identified factors that would encourage participation such as: having a clear purpose; low participant burden; and being well-managed among others.

**Conclusions:**

We report the first examination and synthesis of stakeholder perspectives on registries broadly with a specific focus on neurological patient registries. The findings of the broad literature review were congruent with the neurological patient and caregiver focus groups. We report common themes across the literature and the focus groups performed. Stakeholder perspectives need to be considered when designing and operating patient registries. Emphasizing factors that promote participation and mitigating barriers may enhance patient recruitment.

## Background

Patient registries represent a well established methodology for prospective data collection with a wide array of applications for clinical research and health care administration [1]. In contrast to randomized controlled clinical trials, patient registry data is often highly generalizable to the source patient population and provides a complimentary mechanism to derive evidence for clinical decision-making and management [2]. Some neurological conditions are sufficiently uncommon or rare that single centre observational studies and randomized controlled clinical trials are unfeasible and thus are good candidates for studies through patient registries. Data sources for patient registries range from clinic-based through administrative data collection and often there is capture of patient demographic and/or medical data. As part of the Public Health Agency of Canada’s National Population Health Study of Neurological Conditions, we undertook the development of Neurological Registry Best Practice Guidelines for Canadian registries [3]. A key aspect of successful guideline development is the incorporation of various stakeholder perspectives to ensure relevance and feasibility. We examined perspectives about registries through a literature review. We subsequently performed neurological patient and caregiver focus groups to further examine patient perspectives about registries. The aim of this study was to conduct a “first-look” at the stakeholder perspectives of patient registries to summarize our understanding of the literature and to conduct focus groups in order to assess relevance to neurological patients in our region.

## Methods

### Literature review

**Figure 1:**
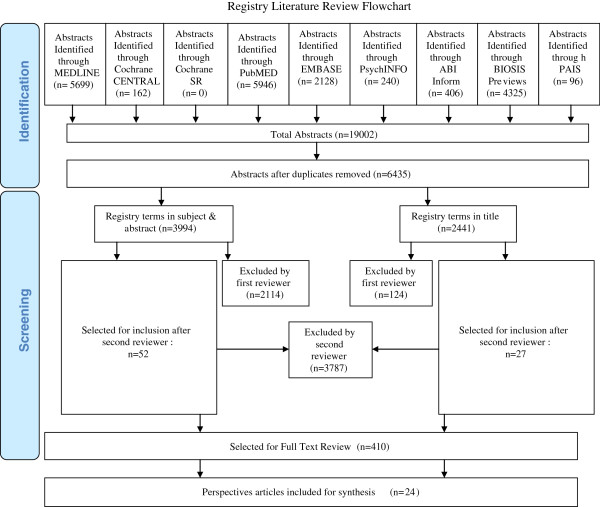
Registry literature review flowchart.

A literature review aiming to identify all patient registry-related literature was performed using search terms such as register, registry and registries. The search strategy (see Additional file 1) was developed in consultation with an experienced research librarian (D.L.) and included the following databases: MEDLINE, EMBASE, PubMED, the Cochrane Library, PsycINFO, ABI Inform, BIOSIS Previews, and PAIS. The flow of article identification and screening is summarized in Figure 1.

Duplicate citations and non-English articles were removed. Rather than apply strict inclusion and exclusion criteria to abstracts reviewed we applied two broad inclusion criteria: 1) the article pertained to patient registries; and 2) the article reported perceptions about patient registries from any relevant stakeholder. We assessed reviewer abstract selection agreement by pilot-testing these inclusion criteria on a sample of 100 abstracts. We then employed a consecutive reviewer method whereby one reviewer excluded clearly irrelevant abstracts followed by a second reviewer who then re-screened the relevant and possibly relevant abstracts for inclusion in the secondary review. Relevant articles underwent full text review. Articles relevant to perspectives regarding patient registries were abstracted for relevant details using Microsoft Excel 2007 (Redmond, Washington) and a descriptive summary was generated. Summaries were synthesized qualitatively based upon relevance to patient registries (Additional file 2).

### Focus groups

#### Recruitment and data collection

Focus group participants were recruited through neurology clinics in Calgary, Canada through physician referrals. A purposive sampling strategy was used, with the goal of recruiting a variety of people living with neurological conditions and their caregivers/parents who would be able to actively participate. Exclusion criteria included developmental delay, cognitive or language impairment that would preclude active participation in the focus group discussions. A one-page information sheet about the research project and the purpose of the focus groups, along with the synthesized literature review was provided to the focus group leaders as background for discussion.

Three focus groups were conducted in April 2012 led by experienced focus group facilitators (G.M., L.C.) and included: 1) parents of children with neurological conditions; 2) patients with dystonia, epilepsy or multiple sclerosis and their caregivers; and 3) patients living with amyotrophic lateral sclerosis (ALS), Huntington’s disease or Parkinson’s disease and their caregivers. The composition of the groups was designed to facilitate common themes and open conversation among the participants (i.e. common themes between caregivers of adult neurodegenerative conditions versus common themes between caregivers of children with neurological conditions).

Each focus group was 90 minutes in length. Each participant received a $10 honorarium to contribute to travel and parking costs. The questions used to guide the focus group discussion are outlined in Table 1.

**Table 1:** Focus group questions

									1.																																																																																Round-table introductions, including: Why were you interested in coming out to this focus group tonight?
									2.																																																																																Generally, what are your thoughts about patient registries?
									3.																																																																																Why might you/your family member be interested in participating in a registry?
									4.																																																																																What might concern you about participating in a registry?
									5.																																																																																What are your thoughts about this information and how it is shared (i.e., information included in a worksheet handout)?
									6.																																																																																What words of advice would you give to doctors and other health professionals about inviting patients to participate in a registry?
																																																																																									Is there anything else you would like to say?

Part-way through the focus group sessions, just before question 4 in Table 1, each participant was provided a worksheet outlining the kinds of information that might be collected by a registry. The focus group participants were given a few minutes to complete the provided worksheet, before entering into group discussion about the kinds of information they were comfortable sharing, the kinds they would be less comfortable sharing and why. Participants were asked about other kinds of information (i.e., not included on the worksheet) that could be collected through registries. The unidentified completed worksheets were collected at the end of the focus groups with each participant’s consent.

#### Data management and analysis

The focus groups were audio-taped and transcribed, with back-up notes taken. Using constant comparative analysis, transcripts and notes were reviewed with the purpose of identifying key themes relative to the focus group questions. Constant comparative analysis is interpretational and theory building, and involves moving back and forth between data collection and analysis [4]. The two analysts (G.M. & L.C.) did preliminary analysis of the data collected after each focus group, and then used these preliminary themes to inform the questioning in subsequent focus groups. More in-depth analysis and interpretation as the focus groups progressed involved looking for both similarities and differences, within and between focus groups, with the goal of identifying key themes as well as the relationships between them. Data management and analysis was facilitated through the use of mind-mapping software, MindJet, San Francisco, California).

#### Ethics approval

Due to the involvement of patients, families and caregivers in the focus group portion of the project, ethics approval was obtained from the University of Calgary’s Conjoint Health Research Ethics Board and the Public Health Agency of Canada Ethics Review Board. All focus group participants provided their informed consent prior to the commencement of the focus group.

## Results

### Literature review

We identified 19,002 abstracts with 6,435 remaining after duplicates were removed as summarized in Figure 1. The first reviewer excluded 2,238 abstracts with an additional 3,787 subsequently excluded by the second reviewer. Full text review was performed on 410 articles. A total of 24 articles were included in the final synthesis. Identified stakeholders from the literature review included registry participants (i.e. patients), clinical care providers (treating physicians often in possession of medical data), research ethics boards, and data users (researchers, governmental agencies, health medical organizations).

#### Participants

In general, most participants have an understanding of the purpose and nature of registries and are in favour of them [5-9]. Motivating factors for participation in registries included the importance of altruism, use of data for legitimate purposes by responsible people, advancement of research that improves the possibility of a treatment or cure among other factors (see Table 2).

**Table 2:** Motivating factors for patient participation in registries

				1.																																																																																										Altruistic attitudes – the perception of benefit to the greater good even beyond immediate individual benefit or the potential for individual benefit [10,16]
				2.																																																																																										That data will be used by responsible people for legitimate purposes – participants desire clear purposes for collecting data and clear methods for its release [10]
				3.																																																																																										Advancement in research and the possibility of elucidation of treatment or cure, [11] and subsequently improved quality of life [16]
				4.																																																																																										Desire for prompt information after diagnosis [7]
				5.																																																																																										Perception of equal communication with health practitioners and researchers [7]
				6.																																																																																										Other factors influencing participation include satisfaction with care, [13] age, education, gender and recruiting site [12,22]

*Identified barriers to registry participation* included: (1) concerns about privacy – particularly around the risk of data falling into the hands of employers especially for current and former health sector clients; [10] (2) concerns about additional visits particularly physical visits as well as associated transportation and financial cost [6,11,12]. Concerns regarding privacy were a strong predictor of willingness to participate in a registry [13]. However, many participants were unconcerned about the inclusion of identifiers in the registry, particularly if it facilitated research contact [14].

With respect to registry services participants have a strong desire for information including educational outreach activities, [15] and up to date discussion of the latest prevention, treatment and disease research, [16] especially if tailored to individual needs or disease sub-types, [7] however there is a clear preference for contact with a known provider over registry personnel [15]. Toll-free assistance services, [15] and other similar initiatives may thus be a poor use of limited resources. There was a desire from registry participants to see regular communication of results (e.g. annual reports, newsletters) in lay language, [7] however again while it was preferred that these were interactive, sophisticated technologies such as videos were not preferred [7]. In some disease audiences there was a desire for support and services (e.g. equipment) as well as family support especially for siblings of affected children [7].

*Clinical care providers* are motivated to participate in a registry project if burden is minimal, data entry is efficient and simple, [17] operation is low cost, [18] and results or outcomes are relevant to clinical practice or research interests [19,20]. Additionally there is a strong desire to see registry data be freely exchanged and comparable between departments, regions, and countries, [5,18] and online registries help to facilitate this [17]. Finally, provider input at all levels of registry operation is a key aspect of success [9,19,21]. Where physicians are asked to provide their consent prior to contacting their patients for a registry there was some evidence that this interfered with patient recruitment. In one study, there were noticeable differences in physicians refusing patient contact between male (1.3%) and female (4.3%) patients [22].

A significant inhibitor of clinical care provider participation is mandatory participation due to the perception that they would be forced to participate in research that was not relevant to their care or practice or research interests [20]. With respect to registry services, clinical care providers were generally in favor of activities such as educational outreach [15]. Overall, early care provider engagement in registries can provide an opportunity to develop a collaborative spirit among clinical care providers and can be utilized as a tool to inform and standardize clinical practice [19].

#### Data users

There was limited discussion of researcher or industry perceptions regarding registries. One study, [23] did assess the perceptions of research teams who had obtained registry data. All of these researchers reported that the registry was very (54%) or somewhat useful (46%) [23]. Similarly a clear majority (69%) also found the registry’s rapid access to health information to be very or somewhat useful. 38% of the research teams reported that they could have met their recruitment targets using the registry as the sole recruitment pathway [23]. The remaining teams reported they would require at least one other pathway. In general this was clearly delineated by the specificity of inclusion criteria for the study. Almost half (46%) of the research teams also reported that utilizing the registry for recruitment had freed up personnel resources for non-recruitment activities with an average savings of 82 hours [23].

#### Patient recruitment

In several studies the majority of patients were in favor of being contacted directly about research opportunities [14,22]. The mechanism of contact between letter or telephone contact was not reported to have a significant preference in any literature. Where patients indicated they desired that their physician be contacted about the research this was a simple notification rather than a request for permission [22].

### Focus groups

A total of 27 individuals participated in the three focus groups (see Table 3).

**Table 3:** Focus group participants

**Focus group participants**	**Neurological condition**	**Role**		**Gender**	
		**Parent/care-giver**	**Person living with neurological condition**	**F**	**M**
**Group A**	Epilepsy (4)	9	0	7	2
**(n = 9)**	Hydrocephalus (1)				
	Muscular dystrophy (1)				
	Tourette Syndrome (3)				
**Group B**	Dystonia (3)	0	8	7	1
**(n = 8)**	Epilepsy (3)				
	MS (2)				
**Group C**	ALS (3)	3	7	3	7
**(n = 10)**	Huntington’s (2)				
	Parkinson’s (5)				
**Total**	27	12	15	17	10

Group A included only parents of children living with neurological conditions.

#### Reasons for/interest in participating in a registry

Participants described a number of reasons why they might be interested in participating in a registry: to help others living with neurological conditions; to develop a “big picture” about a particular condition; to develop ‘best practices’; and to have access to credible, useful information about their condition.

Altruism emerged as an important factor influencing people’s willingness to participate in a registry. Most people were interested in contributing to the generation of new knowledge that will help people living with these conditions. This was also reflected in people’s explanations about their interest in participating in these focus groups.

A number of people liked the idea of having a registry collecting information about the “big picture” of a neurological condition(s) (e.g., incidence, prevalence, natural history of the disease, treatments and outcomes, co-morbidities). There was discussion about the importance of collecting information about co-morbidities in one focus group in particular (e.g., the numbers of people with cerebral palsy who also have epilepsy; the number of people with MS who also have vascular problems).

#### Factors that would influence participation

A number of factors that would influence participation in a patient registry emerged through the focus groups. The main factors discussed were that the registry would need to have:

1. A clear purpose; A number of focus group participants spoke about the importance of the registry having a clear purpose, and that the purpose would need to be clearly articulated to prospective participants. They would also consent to participate in a registry if they had a good understanding of what the registry was being developed for, and why their participation was important.

2. An opportunity to participate in ethical research that will ultimately make a difference to people living with the condition; Some people indicated that they would want to know if pharmaceutical company involvement or funding would be associated with the registry.

Most people stated that they would not want to be directly contacted by researchers asking them to participate in trials, but rather would want the initial invite to come through their neurologist or neurology clinic. The important consideration for a number of people was that the invitation come from someone with whom they had a trusting relationship, and who knew them and their condition well. This sentiment was particularly strongly expressed in Group B. Many of the Group C focus group participants, however, said they didn’t mind being contacted directly by researchers. This was most strongly expressed by some of the ALS patients.

3. Appropriate management and sustainability; Participants discussed wanting assurance that the registry was well managed and likely to be sustainable before consenting to participate.

Appropriate participant burden; Some people noted that the commitment required of them would influence their interest in participating in a registry. Once again, the time people would be willing to commit would be influenced by their view on the value of the registry. A few people specifically said that they would require an option to withdraw their participation at any time before they would consent to participate in a registry.

#### Types of information that people are concerned about sharing

Overall, the majority of people would be happy to share medical and health information when they understand how collecting this information helps to advance knowledge of a condition, improve treatments, etc. People expressed far more comfort in sharing their medical information than their personal information (i.e., information that might identify them).

#### Privacy and security

People did not want to have their personal information (e.g., name, address, phone number, email, etc.) connected with their medical information. Generally speaking, focus group participants were quite comfortable with appropriate sharing of anonymised, aggregate medical and health information collected by a registry.

Focus group participants described the onus being on the registry to keep the information private, with no ability to connect any personal identifying information with their medical information. The security provisions in a patient registry would have to be excellent, and there would need to be a clear security protocol in place around the handling, sharing and disposing of information.

#### Sharing of information and knowledge

Many participants discussed the importance of ensuring that the knowledge generated through a registry is disseminated. There was some tension between protecting privacy while ensuring that access to registry information by people with a legitimate need or interest is maximized. Privacy and confidentiality were felt to be important, though some people realized that there needed to be some kind of balance as too much emphasis on confidentiality contributes to other problems. Many [but not all] people want anonymized medical/health information widely shared if it can assist in the generation of useful knowledge.

In all focus groups a specific question was asked about whether people would be concerned with registry information being transferred to other universities if any identifying information such as name and address were removed. None of these focus group participants expressed concern about the sharing of anonymized data with other academic centres.

#### Inviting patients to participate in a registry

The majority of participants indicated that they would prefer an invitation from their doctor, and preferably their specialist or someone in the neurology clinic. In some cases this could be a nurse manager or someone else affiliated with the clinic. Most people prefer a personal, individualized approach from someone they know and trust, and who knows them.

Most focus group participants said they preferred a personal invitation to participate either over the phone or face-to-face as this format provides opportunities to ask questions. Another option described by some would be to get a personal letter in the mail from someone you know and trust, which could be followed up with a meeting and/or phone call.

#### Recruitment at time of diagnosis

A number of individuals said that it’s generally not a good idea to approach someone about participating in a registry when they are newly diagnosed. The timing post-diagnosis was thought to vary from person to person, with people suggesting that “*your medical team knows when you are ready*, *knows where you are at*.”

## Discussion

We performed a comprehensive review of the literature pertaining to stakeholder perspectives on patient registries to determine the current state of understanding. Due to the lack of previous reviews and the large number of publications pertaining to patient registries this review was designed as a comprehensive exploratory rather than standard systematic review method. This method enabled the inclusion of studies that would have been excluded if extensive inclusion and exclusion criteria had been applied. The findings of the literature review was not limited to neurological registries (Additional file 3), but rather is based upon the literature relating to any disease. To investigate the relevance to neurological conditions focus groups were conducted with patients with neurological conditions and their caregivers.

Overall, both the literature review and focus groups support that patients carefully consider registry goals and operations when deciding whether or not to participate. Patients expect their information to be managed appropriately and that the project has a reasonable chance of resulting in beneficial findings. Patients with more severe conditions (i.e. ALS) appear to have less reluctance about sharing their medical information. This latter finding may reflect a sense of urgency for research to develop meaningful treatment options in these more severely affected patients.

The literature review identified perceptions that should be important considerations for designing, implementing and operating patient registries. From a patient registry participant perspective the literature supports altruism, responsible use of data and advancement of research among others as motivating factors for participating in a patient registry. Barriers to participation included concerns about privacy and participant burden (i.e. extra clinic visits and associated costs). Importantly, a desire to see regular communication of results was cited. Motivating factors for clinical care providers included minimal burden, efficient and simple data entry, low operation cost and relevance of results or outcomes to their practice or research. Researchers and other data users reported patient registries to be a generally useful source of data and as a method of patient recruitment for clinical studies. Consideration of these motivating factors and barriers should be given to maximize patient registry interactions with these groups. Registry participants reported a desire for their care provider to be notified upon enrollment, a process that can be readily incorporated into registries.

We subsequently conducted focus groups including patients and caregivers across the spectrum of neurological conditions to obtain their perspectives about registries and specific data that may be collected. These focus groups re-iterated some of the themes identified in the literature review. The focus group participants agreed that in order for them to participate a registry requires a clear purpose. Patients do view participation as an opportunity to access ethical research that will make a difference to people. Participant burden is a factor that in part determines willingness to participate. Patients feel that they should be able to withdraw from the registry at any time. While patients expressed more concerns about sharing personal data than medical data, the relevance of the data to the overall aim of the registry was a strong factor in determining whether their data should be provided or not. Some differences in the extent to which focus group participants would consider sharing data were observed with caregivers of affected children being more reluctant and patients with ALS being less reluctant to share data. Overall, findings from focus groups with patients with neurological conditions and their caregivers suggest that motivations for this group are similar to those found in a literature review of patient registries in general.

The findings are useful for the development of best practices. Best practices must consider enabling factors and barriers to registry development and operations. Consideration of stakeholder perspectives is essential to success. As an example, our focus groups indicate that patients with neurological conditions and their caregivers may not be willing to provide social insurance numbers (SIN). Developing a registry with administrative data linkage based on SIN may not be feasible in our region based on these results.

Strict limitations need to be considered when applying the findings of this study. The literature review did not employ a “systematic” review methodology increasing the possibility that a single reviewer did not include a relevant article. We expect that this is unlikely given the inclusive design of the search strategy and liberal inclusion of articles into the full text review stage. However, this review did not include non-English articles or survey the grey literature. Limitations for the focus group method include the small number of participants from each disease group. However, the purpose of the focus groups was to obtain commonalities in the perspectives of patient registries across the spectrum of adult and pediatric neurological conditions and the participants in the focus groups were representative of that aim. Patient perspectives are likely to vary with geographic, cultural and socioeconomic differences.

## Conclusions

With increasing recognition that patient registries represent a valid, effective and important methodology for the collection of prospective observational data and the continued emergence of new patient registries for neurological conditions, it is essential to consider the perspectives of all relevant stakeholders. Strategies to motivate participants, caregivers, stakeholders, governmental and administrative bodies as well as the research community are instrumental to successful registry outcomes. This study examined patient and caregiver perspectives across the available literature and compared them to those identified in our local focus groups finding them to be highly consistent. Future studies should examine consistency of these findings in other regions with differing cultural norms and health care systems.

## Abbreviations

ALS: Amyotrophic lateral sclerosis; MS: Multiple sclerosis; PAIS: Public affairs information service.

## Competing interests

The authors declare that they have no competing interests.

## Authors’ contributions

LK co-lead the development of the project, assisted with the literature review and lead the authorship of the manuscript. GM designed, conducted, analyzed the focus groups and contributed to the authorship of the manuscript. LC designed, conducted, analyzed the focus groups and contributed to the authorship of the manuscript. MJ co-lead the development of the project, assisted with the literature review and contributed to the authorship of the manuscript. LD assisted with the literature review and contributed to the authorship of the manuscript. DL assisted with the literature review and contributed to the authorship of the manuscript. DL assisted with the design and testing of the search strategy and contributed to the authorship of the manuscript. JW assisted with the literature review and focus groups and contributed to the authorship of the manuscript. NJ co-led the development of the project, led the literature review and contributed to the authorship of the manuscript. TP co-led the development of the project, led the focus groups and contributed to the authorship of the manuscript. All authors read and approved the final manuscript.

## Pre-publication history

The pre-publication history for this paper can be accessed here:

http://www.biomedcentral.com/1471-2288/13/135/prepub

## Supplementary Material

Additional file 1 Supplementary Data: Search Strategy. Click here for file

Additional file 2 Patient registries. Click here for file

Additional file 3 My Thoughts on the Types of Information Neurological Registries Could Collect. Click here for file
