# Supplementary material for: Perspectives on neurological patient registries: a literature review and focus group study
Source: BMC Med Res Methodol. 2013 Nov 9;13:135. doi: 10.1186/1471-2288-13-135 (PMC4225768; doi:10.1186/1471-2288-13-135)
Supplement: Additional file 1 — Supplementary Data: Search Strategy. [file 1471-2288-13-135-S1.doc]

**Additional file 1 Supplementary Data: Search Strategy**

**MEDLINE**

**Cochrane CENTRAL Register of Controlled Trials**

**Cochrane Database of Systematic Reviews**

1. exp *Registries/

2. ((patient or patients or disease* or population surveillance) adj5 (registry or registries or register or registers)).tw.

3. 1 or 2

4. exp Population Surveillance/mt [Methods]

5. Data Collection/lj, mt, og, st [Legislation & Jurisprudence, Methods, Organization & Administration, Standards]

6. models, theoretical/ or models, organizational/

7. "organization and administration"/ or exp governing board/ or knowledge management/ or mandatory programs/ or organizational objectives/ or planning techniques/ or program development/ or public health administration/ or total quality management/ or voluntary programs/

8. Accreditation/

9. "Forms and Records Control"/

10. Benchmarking/

11. (barrier* or best practice* or creating or creation or design* or develop or developing or development or establish* or evolving or evolution or facilitator* or guidelines or implementing or implementation or infrastructure* or lessons learned or maintenance or maintain* or methodology or model or models or quality or standards or trends).tw.

12. 4 or 5 or 6 or 7 or 8 or 9 or 10 or 11

13. 3 and 12

14. exp *Registries/mt, og, st [Methods, Organization & Administration, Standards]

15. 13 or 14

16. limit 15 to english

17. limit 16 to animals

18. limit 16 to (animals or humans)

19. 17 not 18

20. 16 not 19

21. limit 20 to (comment or editorial or letter)

22. 20 not 21

23. exp *Tissue Donors/ or exp *"Tissue and Organ Procurement"/

24. ((organ or organs or tissue*) adj5 (donor* or donat*)).tw.

25. education.fs.

26. exp "substance-related disorders"/

27. exp "drug and narcotic control"/

28. exp street drugs/

29. ((drug or drugs or pharmaceutical*) adj5 (registry or registries or register or registers)).tw.

30. exp vital statistics/

31. ((marriage or birth or death) adj5 (registry or registries or register*)).tw.

32. exp *vaccination/ or exp *immunization/

33. (immunisation* or immunization* or vaccination* or vaccine*).ti.

34. (cochrane adj5 register).tw.

35. 23 or 24 or 25 or 26 or 27 or 28 or 29 or 30 or 31 or 32 or 33 or 34

36. 22 not 35

**PubMED**

1. Registries[Majr]

2. ((patient or patients or disease* or population surveillance) AND (registry or registries or register or registers))[tiab]

3. 1 or 2

4. Population Surveillance/mt [Methods][MeSH]

5. Data Collection/lj, mt, og, st [Legislation & Jurisprudence, Methods, Organization & Administration, Standards][MeSH]

6. models, theoretica[MeSH] or models, organizational[MeSH]

7.("organization and administration" or governing board or knowledge management or mandatory programs or organizational objectives or planning techniques or program development or public health administration or total quality management or voluntary programs)[MeSH]

8. Accreditation[MeSH]

9. "Forms and Records Control"[MeSH]

10. Benchmarking[MeSH]

11. (barrier* or best practice* or creating or creation or design* or develop or developing or development or establish* or evolving or evolution or facilitator* or guidelines or implementing or implementation or infrastructure* or lessons learned or maintenance or maintain* or methodology or model or models or quality or standards or trends)[tiab]

12. 4 or 5 or 6 or 7 or 8 or 9 or 10 or 11

13. 3 and 12

14. Registries/mt, og, st [Methods, Organization & Administration, Standards][Majr]

15. 13 or 14

16. limit 15 to english

17. limit 16 to animals

18. limit 16 to (animals or humans)

19. 17 not 18

20. 16 not 19

21. limit 20 to (comment or editorial or letter)

22. 20 not 21

23. Tissue Donors[Majr] or "Tissue and Organ Procurement"[Majr]

24. ((organ or organs or tissue*) and (donor* or donat*))[tiab]

25. education[fs]

26. "substance-related disorders"[MeSH]

27. "drug and narcotic control"[MeSH]

28. street drugs[MeSH]

29. ((drug or drugs or pharmaceutical*) and (registry or registries or register or registers))[tiab]

30. vital statistics[MeSH]

31. ((marriage or birth or death) and (registry or registries or register*))[tiab]

32. vaccination[Majr] or immunization[Majr]

33. (immunisation* or immunization* or vaccination* or vaccine*)[ti]

34. (cochrane register)[tiab]

35. 23 or 24 or 25 or 26 or 27 or 28 or 29 or 30 or 31 or 32 or 33 or 34

36. 22 not 35

**EMBASE**

1. *register/

2. ((patient or patients or disease* or population surveillance) adj5 (registry or registries or register or registers)).tw.

3. 1 or 2

4. *model/ or *process model/

5. *"organization and management"/

6. *management/ or *total quality management/

7. *planning/ or *strategic planning/

8. *organization/

9. methodology/ or *quality control/ or standard/

10. data collection method/

11. *quality control/

12. *"board of trustees"/

13. *knowledge management/

14. *program development/

15. *planning/

16. 4 or 5 or 6 or 7 or 8 or 9 or 10 or 11 or 12 or 13 or 14 or 15

17. 3 and 16

18. ((barrier* or best practice* or creating or creation or design* or develop or developing or development or establish* or evolving or evolution or facilitator* or guidelines or implementing or implementation or infrastructure* or lessons learned or maintenance or maintain* or methodology or model or models or quality or standards or trends) adj5 (registry or register or registers or registries) adj5 (patient or patients or disease* or surveillance)).tw.

19. 17 or 18

20. limit 19 to english language

21. limit 20 to animal studies

22. 20 not 21

23. limit 22 to (conference abstract or "conference review" or editorial or letter or note or proceeding or report or trade journal)

24. 22 not 23

25. exp *donor/

26. exp *transplantation/

27. ((organ or organs or tissue*) adj5 (donor* or donat*)).tw.

28. exp *drug dependence/ or exp *drug abuse/ or exp *substance abuse/ or exp *alcoholism/ or exp *addiction/

29. exp *drug control/

30. ((drug or drugs or pharmaceutical*) adj5 (registry or registries or register or registers)).tw.

31. exp *birth certificate/

32. exp *death certificate/

33. exp *"cause of death"/ or exp *marriage/

34. exp *vital statistics/

35. ((marriage or birth or death) adj5 (registry or registries or register*)).tw.

36. exp *vaccination/

37. exp *immunization/

38. (immunisation* or immunization* or vaccination* or vaccine*).tw.

39. (cochrane adj5 register).tw.

40. 25 or 26 or 27 or 28 or 29 or 30 or 31 or 32 or 33 or 34 or 35 or 36 or 37 or 38 or 39

41. 24 not 40

**PsycINFO**

1. ((barrier* or best practice* or creating or creation or design* or develop or developing or development or establish* or evolving or evolution or facilitator* or guidelines or implementing or implementation or infrastructure* or lessons learned or maintenance or maintain* or methodology or model or models or quality or standards or trends) adj5 ((patient or patients or disease* or population surveillance) adj5 (registry or registries or register or registers))).tw.

2. ((organ or organs or tissue*) adj5 (donor* or donat*)).tw.

3. ((drug or drugs or pharmaceutical*) adj5 (registry or registries or register or registers)).tw.

4. ((marriage or birth or death or vital statistics) adj5 (registry or registries or register*)).tw.

5. (immunisation* or immunization* or vaccination* or vaccine*).ti.

6. (cochrane adj5 register*).tw.

7. 2 or 3 or 4 or 5 or 6

8. 1 not 7

9. limit 8 to english language

10. limit 9 to (abstract collection or chapter or "column/opinion" or "comment/reply" or dissertation or editorial or letter or review-book or review-media or review-software & other)

11. 9 not 10

**ABI Inform**

**BIOSIS Previews**

**PAIS (Public Affairs Information Service)**

1. ((patient or patients or disease* or population surveillance) and (registry or registries or register or registers))[Keyword]

2. (barrier* or best practice* or creating or creation or design* or develop or developing or development or establish* or evolving or evolution or facilitator* or guidelines or implementing or implementation or infrastructure* or lessons learned or maintenance or maintain* or methodology or model or models or quality or standards or trends)[Keyword]

3. 1 and 2

4. ((organ or organs or tissue*) and (donor* or donat*))[Keyword]

5. ((drug or drugs or pharmaceutical*) and (registry or registries or register or registers))[Keyword]

6. ((marriage or birth or death) and (registry or registries or register*))[Keyword]

7. (immunisation* or immunization* or vaccination* or vaccine*)[Keyword]

8. (cochrane register)[Keyword]

9. 4 or 5 or 6 or 7 or 8

10. 3 and 9
