# Supplementary material for: Perspectives on neurological patient registries: a literature review and focus group study
Source: BMC Med Res Methodol. 2013 Nov 9;13:135. doi: 10.1186/1471-2288-13-135 (PMC4225768; doi:10.1186/1471-2288-13-135)
Supplement: Additional file 2 — Patient registries. [file 1471-2288-13-135-S2.pptx]

## Slide 1
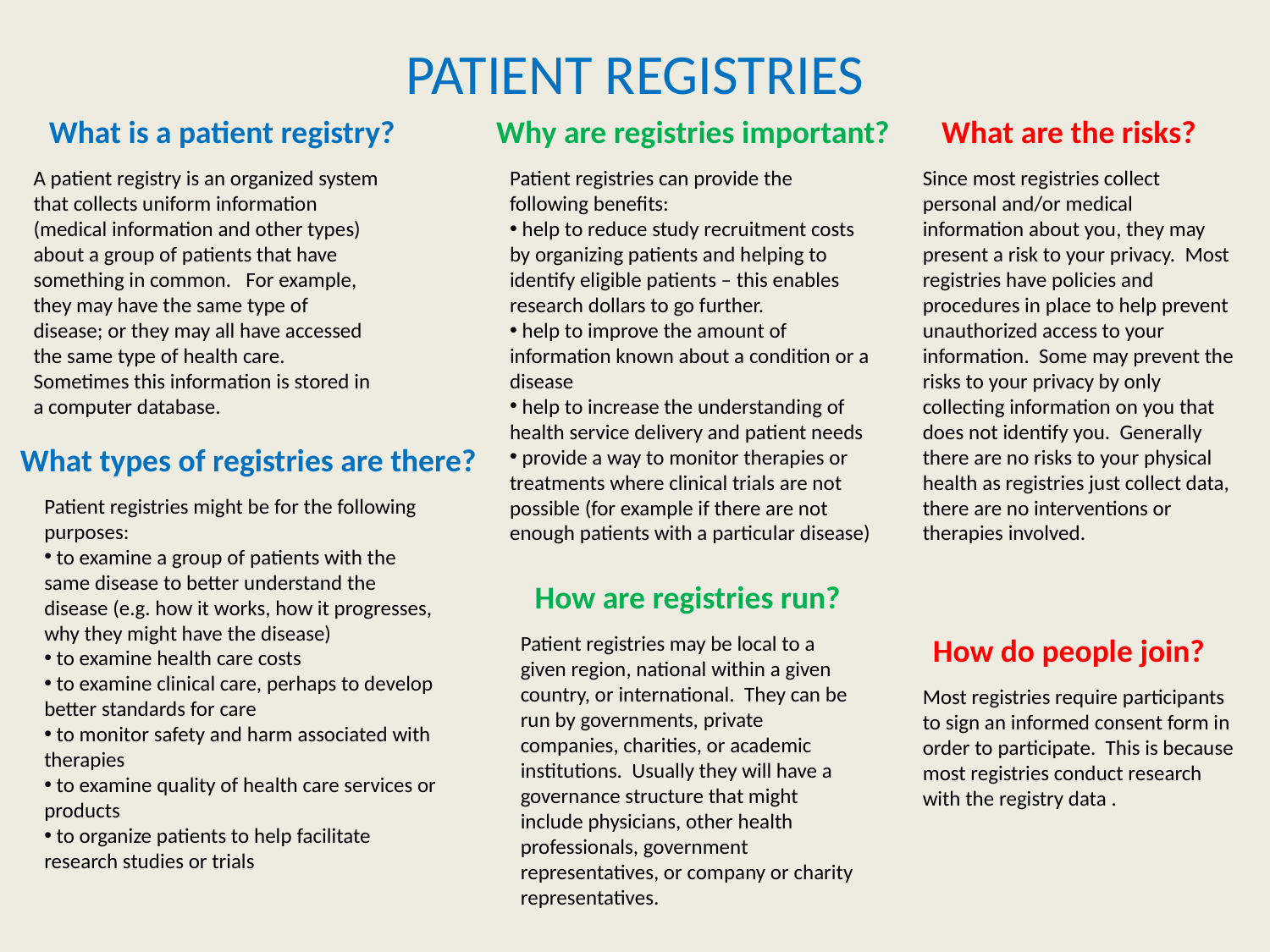

PATIENT REGISTRIES
What is a patient registry?
Why are registries important?
What are the risks?
A patient registry is an organized system that collects uniform information (medical information and other types) about a group of patients that have something in common. For example, they may have the same type of disease; or they may all have accessed the same type of health care. Sometimes this information is stored in a computer database.
Patient registries can provide the following benefits:
 help to reduce study recruitment costs by organizing patients and helping to identify eligible patients – this enables research dollars to go further.
 help to improve the amount of information known about a condition or a disease
 help to increase the understanding of health service delivery and patient needs
 provide a way to monitor therapies or treatments where clinical trials are not possible (for example if there are not enough patients with a particular disease)
Since most registries collect personal and/or medical information about you, they may present a risk to your privacy. Most registries have policies and procedures in place to help prevent unauthorized access to your information. Some may prevent the risks to your privacy by only collecting information on you that does not identify you. Generally there are no risks to your physical health as registries just collect data, there are no interventions or therapies involved.
What types of registries are there?
Patient registries might be for the following purposes:
 to examine a group of patients with the same disease to better understand the disease (e.g. how it works, how it progresses, why they might have the disease)
 to examine health care costs
 to examine clinical care, perhaps to develop better standards for care
 to monitor safety and harm associated with therapies
 to examine quality of health care services or products
 to organize patients to help facilitate research studies or trials
How are registries run?
Patient registries may be local to a given region, national within a given country, or international. They can be run by governments, private companies, charities, or academic institutions. Usually they will have a governance structure that might include physicians, other health professionals, government representatives, or company or charity representatives.
How do people join?
Most registries require participants to sign an informed consent form in order to participate. This is because most registries conduct research with the registry data .
