# Supplementary material for: Perspectives on neurological patient registries: a literature review and focus group study
Source: BMC Med Res Methodol. 2013 Nov 9;13:135. doi: 10.1186/1471-2288-13-135 (PMC4225768; doi:10.1186/1471-2288-13-135)
Supplement: Additional file 3 — My Thoughts on the Types of Information Neurological Registries Could Collect. [file 1471-2288-13-135-S3.docx]

**My Thoughts on the Types of Information Neurological Registries Could Collect**

Focus Group Date:

Neurological Condition:

**Age:**

Imagine you have chosen to participate in a patient registry for your (or your family member’s) condition: If you would be willing to share the type of information below in a patient registry place a checkmark (✓) next to the item. If you would not be willing to share the type of information below in a patient registry place an “X” next to the item. You will not be asked to provide information as part of this project. We are just interested in better understanding the types of information that are important to you.

Name

Address

Phone number

Email Address

Household Income

Highest level of education

Occupation

Marital status

Healthcare number

Social insurance number

Date of Birth

Place of Birth

Gender

Ethnicity

Disease type

Height

Weight

Preferred language for communication

Clinic details (your physician’s information, name of the clinic etc.)

Genetics (results of a genetic test if performed)

Heart tests (ECG, echocardiogram, MRI, cardiac function tests, blood pressure tests)

Lung tests (pulmonary function tests, x-rays, blood gas levels)

Information on mobility (including the use of assistance devices such as canes, walkers and wheelchairs)

Information on your diet (including information on your ability to swallow or if you have a feeding tube)

Information on your mood

Muscle biopsy results

Smoking history

Information on your medications

Lung therapies (including Bi-PAP and other types of ventilation)

Study participation (whether or not you have participated in studies)

Other medical conditions (diabetes, high blood pressure, major surgeries or trauma, cataracts)

Cognitive abilities (including information on developmental delay and dementia)

Information on your levels of fatigue or sleepiness

Caregiver information (name, contact details, whether they live with you)

Information about how you cope with daily tasks (such as writing, speaking, dressing, personal hygiene)

Information on your personal directive

Participation in charitable groups that support patients with your condition

If your disease is hereditary, information on other affected family members

Tissue samples

Information on the location of tissue samples you have provided

**If there is information you think we should collect that we haven’t listed above, please list it below.**

**If you have any other comments or thoughts, please write them below.**

**Thank you for providing us with this valuable information. We will be taking some time to discuss this during our focus group session today. If you are comfortable doing so, please leave your completed sheet with the focus group facilitator at the end of the session.**
